# *Glaesserella parasuis* infection disrupts the gut–lung axis via microbiota dysbiosis and metabolic reprogramming leading to intestinal barrier impairment in piglets

**DOI:** 10.3389/fcimb.2026.1740531

**Published:** 2026-02-05

**Authors:** Na Li, Aobo Shen, Xinlu Sun, Ying Guo, Ronglan Yin, Meiling Qian, Fanhua Zeng, Haoran Wang, Xueqian Liu, Menglu Li, Yuanyuan Zhou, Ronghuan Yin

**Affiliations:** 1 Key Laboratory of Livestock Infectious Diseases, Ministry of Education, and Key Laboratory of Ruminant Infectious Disease Prevention and Control (East), Ministry of Agriculture and Rural Affairs, College of Animal Science and Veterinary Medicine, Shenyang Agricultural University, Shenyang, China; 2 State Key Laboratory for Diagnosis and Treatment of Severe Zoonotic Infectious Diseases, Key Laboratory of Zoonosis Research, Ministry of Education, Jilin University, Changchun, China

**Keywords:** *Glaesserella parasuis*, microbial dysbiosis, gut-lung axis, metabolomics, arginine biosynthesis, intestinal barrier dysfunction

## Abstract

**Background:**

*Glaesserella parasuis* (*G. parasuis*), is a key respiratory pathogen responsible for Glässer's disease in pigs, characterized by polyserositis, arthritis, and pulmonary lesions. While it disrupts the respiratory microbiota, its impact on the gut-lung axis, a critical pathway for systemic immune and metabolic crosstalk, remains unexplored.

**Methods:**

We established a piglet infection model using the highly virulent G. parasuis strain XX0306 (serotype 5). Systemic effects were investigated through integrated 16S rDNA sequencing of the lung and gut microbiota, complemented by untargeted metabolomics of intestinal contents. We performed histopathological examination and measured serum biomarkers (diamine oxidase and D-lactate) to assess intestinal barrier integrity. Correlation analysis linked microbial shifts to host metabolic alterations.

**Results:**

Infection induced profound dysbiosis in both the lung and gut microbiota. Pulmonary microbial diversity and functional potential declined. Gut dysbiosis featured a loss of beneficial bacteria and enrichment of potential pathogens (e.g., *Streptococcus*, *Campylobacter*, *Desulfovibrio*). Functional prediction indicated significant alterations in 12 gut microbial metabolic pathways, with downregulated amino acid metabolism and upregulated carbohydrate/lipid metabolism and xenobiotic degradation. Metabolomics identified 30 differentially abundant metabolites (e.g., argininosuccinate, liquiritigenin, citrulline), primarily enriched in cytochrome P450-mediated xenobiotic metabolism and arginine biosynthesis. Argininosuccinate levels correlated with pathogenic genera (*Leucobacter*, *Streptococcus*, *Desulfovibrio*). Infected piglets exhibited significant intestinal barrier damage, evidenced by elevated serum diamine oxidase (DAO) and D-lactate (D-LA).

**Conclusion:**

This study demonstrates that *G. parasuis* infection extensively remodels the gut-lung axis microbiota and host metabolome, leading to intestinal barrier impairment. The perturbation of arginine biosynthesis may compromise host immunity. These results provide novel mechanistic insights into the pathogenesis of Glässer's disease.

## Graphical Abstract

**Figure:**
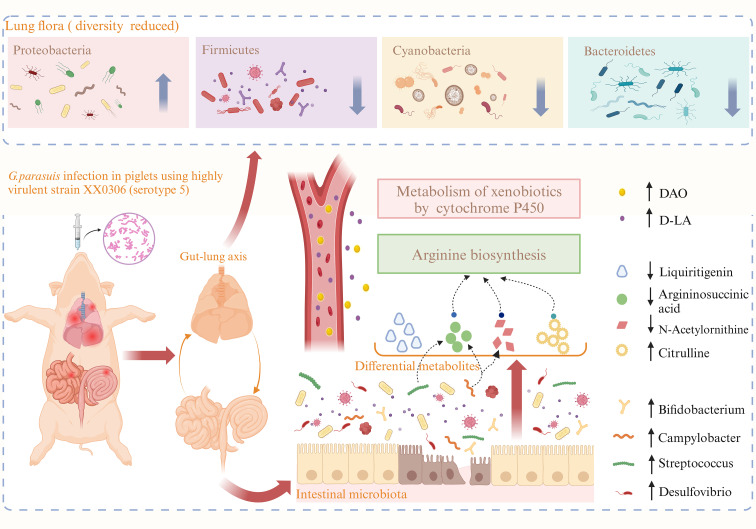

## 1 Introduction

*Glaesserella parasuis* is an important opportunistic pathogen in swine, capable of causing Glässer’s disease under immunosuppressive or stressful conditions (Jia et al., 2025). This disease is characterized by fibrinous polyserositis, arthritis, and severe pneumonia, leading to substantial morbidity and mortality, particularly in weaned piglets, and inflicts considerable economic losses on the swine industry (Dazzi et al., 2020; Gong et al., 2022). Recent advances in microbiomics have shifted focus towards understanding the pathogen’s interaction with the host microbiota. Indeed, previous studies demonstrate that *G. parasuis* infection disrupts the respiratory tract microbiota in piglets, and specific dysbiotic signatures correlate with virulent strain colonization and differential vaccine efficacy (Mahmmod et al., 2020; Obregon-Gutierrez et al., 2025). However, this research has been confined to the respiratory niche, presenting a critical limitation.

Glässer’s disease is a systemic condition with multi-organ involvement, suggesting its pathogenesis likely extends beyond local respiratory ecology. In this context, the communication along the gut-lung axis has emerged as a key frontier. This axis involves complex crosstalk: the gut microbiota orchestrates systemic and pulmonary immunity through the maturation and functional programming of key immune cells (Espirito Santo et al., 2021; Shim et al., 2023; Stevens et al., 2022; Yoo et al., 2020). Conversely, inflammatory signals from the lungs can remodel the gut microbial community, often driving a state of dysbiosis marked by a loss of beneficial symbionts and an expansion of pro-inflammatory taxa, which may compromise colonization resistance and mucosal barrier function (Liu et al., 2024; Sencio et al., 2021; Zhang et al., 2022). Such reciprocal disruption can fuel a vicious cycle of systemic inflammation, potentially exacerbating disease severity (Sencio et al., 2021). Yet, the functional mediators of this axis in *G. parasuis* infection remain uncharacterized. A growing body of evidence indicates that microbiota exert their influence primarily through metabolic intermediates (Dang and Marsland, 2019; Yoon et al., 2023). Microbial metabolites, including short-chain fatty acids, indole derivatives, and bile acids, serve as key signaling molecules (Qayed et al., 2021; Wei et al., 2023; Yu et al., 2024). These metabolites regulate immune homeostasis and mucosal barrier function across distant sites. For example, short-chain fatty acids demonstrate immunomodulatory activity along the gut-lung axis, influencing intestinal permeability and T-cell differentiation (Kim et al., 2013; Park et al., 2015). In bronchiectasis, tauroursodeoxycholic acid produced by Eggerthella lenta impairs pulmonary neutrophil function (Wang et al., 2025). While metabolites such as inosine and short-chain fatty acids modulate anti-tumor immunity and the response to immunotherapy (Zhao et al., 2022). These research confirmed that specific gut-derived metabolites can dictate pathological outcomes in distal organs.

To determine whether *G. parasuis* infection induces gut-lung axis dysbiosis and metabolic dysregulation, we employed an integrated multi-omics approach in a piglet model. By combining 16S rDNA sequencing with untargeted metabolomics, we comprehensively mapped concurrent compositional shifts in gut/lung microbiota and corresponding alterations in the host metabolome. Our study aims to identify the critical microbial metabolites and associated pathways that contribute to the systemic pathogenesis of Glässer’s disease, thereby providing novel functional insights for future therapeutic interventions.

## 2 Materials and methods

### 2.1 Bacterial strain and animal infection model

The highly virulent *G. parasuis* strain XX0306 (serotype 5) was preserved by the Laboratory of the College of Animal Science and Veterinary Medicine, Shenyang Agricultural University. The strain was cultured overnight at 37°C in tryptic soy broth (TSB, Solarbio, Beijing, China) supplemented with 5% fetal bovine serum (Dingguo, Beijing, China) and 2% nicotinamide adenine dinucleotide (NAD, Solarbio).

Twelve four-week-old piglets were purchased from Shuoyang Breeding Cooperative (Xinmin, China). The experimental protocol was approved by the Ethics Committee of Shenyang Agricultural University (No. 202106034). The piglets were randomly divided into two groups of six each and housed under identical conditions. The infection group was intranasally inoculated with 1 × 10^10^ CFU of bacterial suspension, while the uninfected group was administered an equal volume of TSB. All piglets were anesthetized with sodium pentobarbital (30 mg/kg, intravenous injection; Sinopharm Chemical Reagent Co., Ltd., Shanghai, China) and euthanized by exsanguination on day 19 post-infection. A complete necropsy was performed immediately. Gross lesions in major organs (heart, liver, spleen, lungs, kidneys, and intestines) were documented photographically.

### 2.2 Enzyme-linked immunosorbent assay

Blood samples were collected from the anterior vena cava and left at room temperature for 20 minutes to allow natural coagulation, followed by centrifugation at 3,500 rpm for 15 minutes to separate the serum. Serum levels of inflammatory cytokines interleukin 1 beta (IL-1β), interleukin 8 (IL-8), and tumor necrosis factor (TNF-α) were measured using ELISA kits (Enzyme-linked Biotechnology Co. Ltd., Shanghai, China), strictly following the manufacturer’s protocols. Absorbance was measured at 450 nanometers using a microplate reader (Servicebio, Wuhan, China), and a standard curve was generated using the Gen5 software (version 3.11) provided with the instrument to calculate the sample concentration.

Serum markers for intestinal barrier function, diamine oxidase (DAO) and D-lactate, were measured using corresponding ELISA kits (Enzyme-linked Biotechnology Co. Ltd.) following the provided instructions.

### 2.3 Paraffin section preparation and H&E staining

Tissue samples from the heart, liver, spleen, lung, kidney, duodenum, jejunum, ileum, cecum, and colon were collected. Solid organs were trimmed into approximately 1.5 cm × 1.5 cm × 0.5 cm blocks, and intestinal segments were cut to 2 cm in length. All tissues were rinsed with sterile phosphate-buffered saline (PBS, Solarbio), fixed in 4% neutral buffered formalin (Solarbio) for over 24 hours, and processed for paraffin embedding.

The fixed tissues were dehydrated through a graded ethanol series, cleared in xylene (Sinopharm Chemical Reagent Co., Ltd.), embedded in paraffin (Solarbio), and sectioned at 5 μm thickness. Sections were stained with hematoxylin and eosin (H&E) using a commercial kit (Solarbio) and observed under a light microscope (Leica Microsystems, Wetzlar, Germany). Images were captured using Leica Application Suite X software (version 3.7.4).

### 2.4 Immunofluorescence assay

For IFA, deparaffinized and rehydrated sections underwent antigen retrieval by microwave heating in 0.01 M sodium citrate buffer (pH 6.0; Servicebio). Non-specific binding was blocked with 10% goat serum (Solarbio) for 1 h at room temperature. Sections were incubated overnight at 4°C with a rabbit polyclonal anti-*G. parasuis* primary antibody (produced in-house by immunizing rabbits with whole-cell antigens of strain XX0306; diluted 1:500). Following three washes, sections were incubated for 45 minutes at 37°C with a FITC-conjugated goat anti-rabbit IgG secondary antibody (Epizyme, Shanghai, China; diluted 1:200). For effusion samples (pleural, pericardial, and joint fluid), smears were prepared on glass slides, flame-fixed, and processed identically from the blocking step onward. Slides were mounted with 90% glycerol in PBS and visualized under a fluorescence microscope (Leica Microsystems).

### 2.5 Sample collection for microbiome and metabolome analyses

Alveolar lavage fluid (ALF): The entire lung was excised, and bronchoalveolar lavage was performed by instilling and gently kneading with 50 mL of ice-cold sterile PBS. The recovered lavage fluid was centrifuged at 8,000 rpm for 10 min at 4°C. The pellet was snap-frozen in liquid nitrogen and stored at -80°C for subsequent DNA extraction.

Cecal contents: The cecum was exteriorized, and a segment was ligated at both ends. The outer surface was disinfected with 75% ethanol and rinsed with PBS. The segment was opened aseptically, and the luminal contents were collected using a sterile scalpel, placed into 2 mL cryovials, snap-frozen in liquid nitrogen, and stored at -80°C.

### 2.6 16S rRNA gene sequencing and analysis

Microbial DNA was extracted from ALF pellets and cecal contents using the HiPure Stool DNA Kit (Magen, Guangzhou, China) according to the manufacturer’s protocol. The V3-V4 hypervariable region of the bacterial 16S rRNA gene was amplified with universal primers (341F: CCTACGGGNGGCWGCAG-3’; 806R: GGACTACHVGGGTATCTAAT-5’). Amplicons were purified, quantified, and sequenced on an Illumina NovaSeq 6000 platform (PE250) by GeneDenovo Biotechnology Co., Ltd. (Guangzhou, China).

Raw sequencing reads were processed using USEARCH (Version 11.0). After merging, quality filtering, and chimera removal, sequences were clustered into operational taxonomic units (OTUs) at 97% similarity. Taxonomic annotation was performed against the SILVA database (Release 138). Microbial community analysis and identification of differential taxa were performed using the LEfSe method (LDA score > 2.0, P < 0.05). Functional potential was predicted from the 16S data using the Tax4Fun2 package in R (based on KEGG database).

### 2.7 Metabolite extraction and untargeted metabolomics

Metabolites were extracted from cecal content samples by homogenization in 80% methanol-water solution, followed by sonication in an ice-water bath for 6 minutes and centrifugation at 5,000 rpm for 1 minute at 4°C. The supernatant was collected, lyophilized, and reconstituted in 10% methanol. Quality control (QC) samples were prepared by pooling equal volumes from all samples.

Untargeted metabolomic profiling was performed by GeneDenovo Biotechnology Co., Ltd. Specifically, analysis was conducted using ultra-high-performance liquid chromatography coupled with quadrupole-Exactive mass spectrometry (UHPLC-QE-MS) on a Thermo Fisher Vanquish UHPLC system equipped with a Q Exactive HF-X mass spectrometer (Thermo Fisher Scientific, Waltham, MA, USA). Chromatographic separation was achieved on a Hypersil Gold C18 column (100 × 2.1 mm, 1.9 μm; Thermo Fisher Scientific) at 40 °C using a methanol/water gradient in both positive (0.1% formic acid) and negative (5 mM ammonium acetate) ionization modes. Full MS scans ranged from m/z 100 to 1,500. Raw data were processed using Compound Discoverer software (Version 3.3; Thermo Fisher Scientific). Orthogonal partial least squares-discriminant analysis (OPLS-DA) was performed using the ropls package, and the model was validated by permutation tests. Differential metabolites were identified based on a variable importance in projection (VIP) ≥ 1 and a Student’s t-test p-value < 0.05. Metabolite annotation and pathway enrichment analysis were conducted using the KEGG database (https://www.genome.jp/kegg).

### 2.8 Correlation analysis between microbiota and metabolites

Correlations between the relative abundance of differentially abundant intestinal microbial genera and the intensity of differential metabolites were assessed using Pearson’s correlation coefficient, calculated with the cor.test function in R (Version 4.3.1).

### 2.9 Statistical analysis

Data are presented as mean ± standard deviation (SD). Statistical analysis and visualization were conducted using SPSS 15.0 software (SPSS Inc., Chicago, IL, USA) and GraphPad Prism 8.0 software (GraphPad Software, San Diego, CA, USA). Comparisons between two groups were made using an independent samples t-test, with a significance level set at P < 0.05, indicating a statistically significant difference.

## 3 Results

### 3.1 Clinical signs, gross pathology, and pathogen detection following *G. parasuis* infection

**Figure 1:**
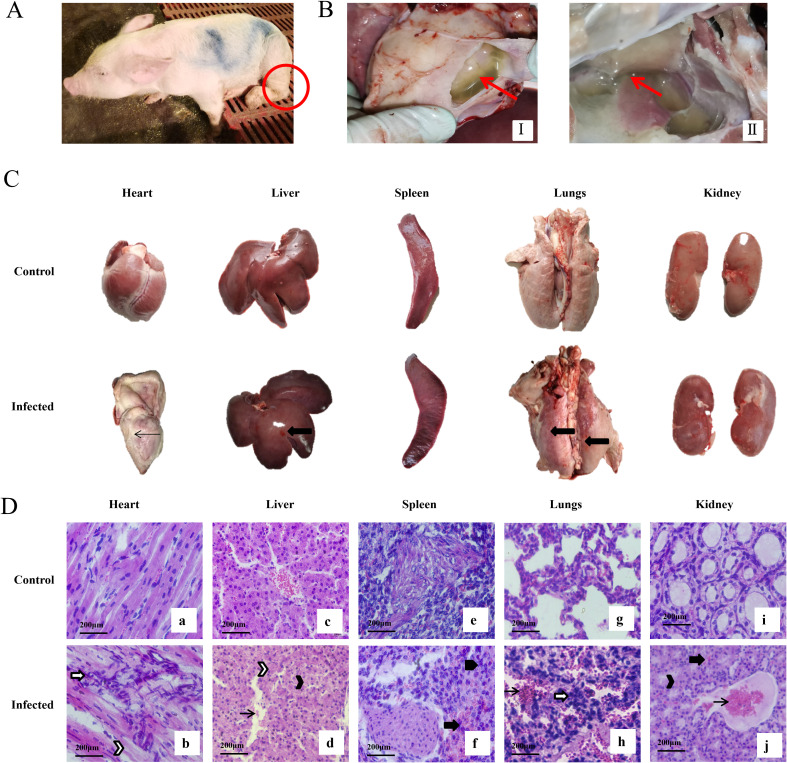
Clinical signs and pathological lesions in piglets caused by infection with *G*. *parasuis*. **(A)** Hind limb swelling in piglets from the infected group. **(B)** Thoracic and pericardial effusions observed in the infected group. I: Pleural effusion; II: Pericardial effusion. **(C)** Pictures of the changes in the dissection of organ. Note: ←: Fibrous exudate; 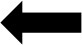: Hemorrhage. **(D)** Pictures of each organ section of piglets(400X). Note: a, c, e, g, and i correspond to the heart, liver, spleen, lungs, and kidneys in the control group; b, d, f, h, and j correspond to the heart, liver, spleen, lungs, and kidneys in the infected group, respectively; 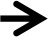: Hyperemia; 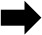: Hemorrhage; 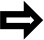: Infiltration of inflammatory cells; 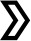: Particle deformation; 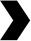: Nuclear lysis; 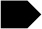: Nuclear condensation. Heart: In the infected group (1-E-b), myocardial fibers exhibited fragmentation and disorganized arrangement, accompanied by granular degeneration and extensive inflammatory cell infiltration. Liver: In the infected group (1-E-d), hepatocytes were disorganized, sinusoids were obliterated, and central venous congestion was observed. Hepatocytes appeared swollen and rounded, with granular degeneration and nuclear dissolution. Spleen: In the infected group (1-E-f), splenic tissue showed hemorrhage, widespread distribution of red blood cells, and significant inflammatory cell infiltration. Lung: In the infected group (1-E-h), alveolar walls were dilated and congested, with extensive inflammatory cell infiltration and loss of alveolar structure. Kidney: In the infected group (1-E-j), nuclear dissolution and congestion were observed, along with swelling and rupture of renal tubules.

Piglets inoculated with *Glaesserella parasuis* strain XX0306 developed clinical signs including growth retardation, coughing, and joint swelling (**Figure 1A**). At necropsy, infected piglets exhibited characteristic lesions of Glässer’s disease: fibrinous polyserositis manifesting as pleural and pericardial effusions with velvet heart appearance, hepatic congestion, and pulmonary consolidation with surface hemorrhages (**Figures 1B, C**). Histopathological analysis confirmed myocardial fiber disruption, alveolar congestion, and extensive inflammatory cell infiltration in the lungs (**Figure 1D**).

**Figure 2:**
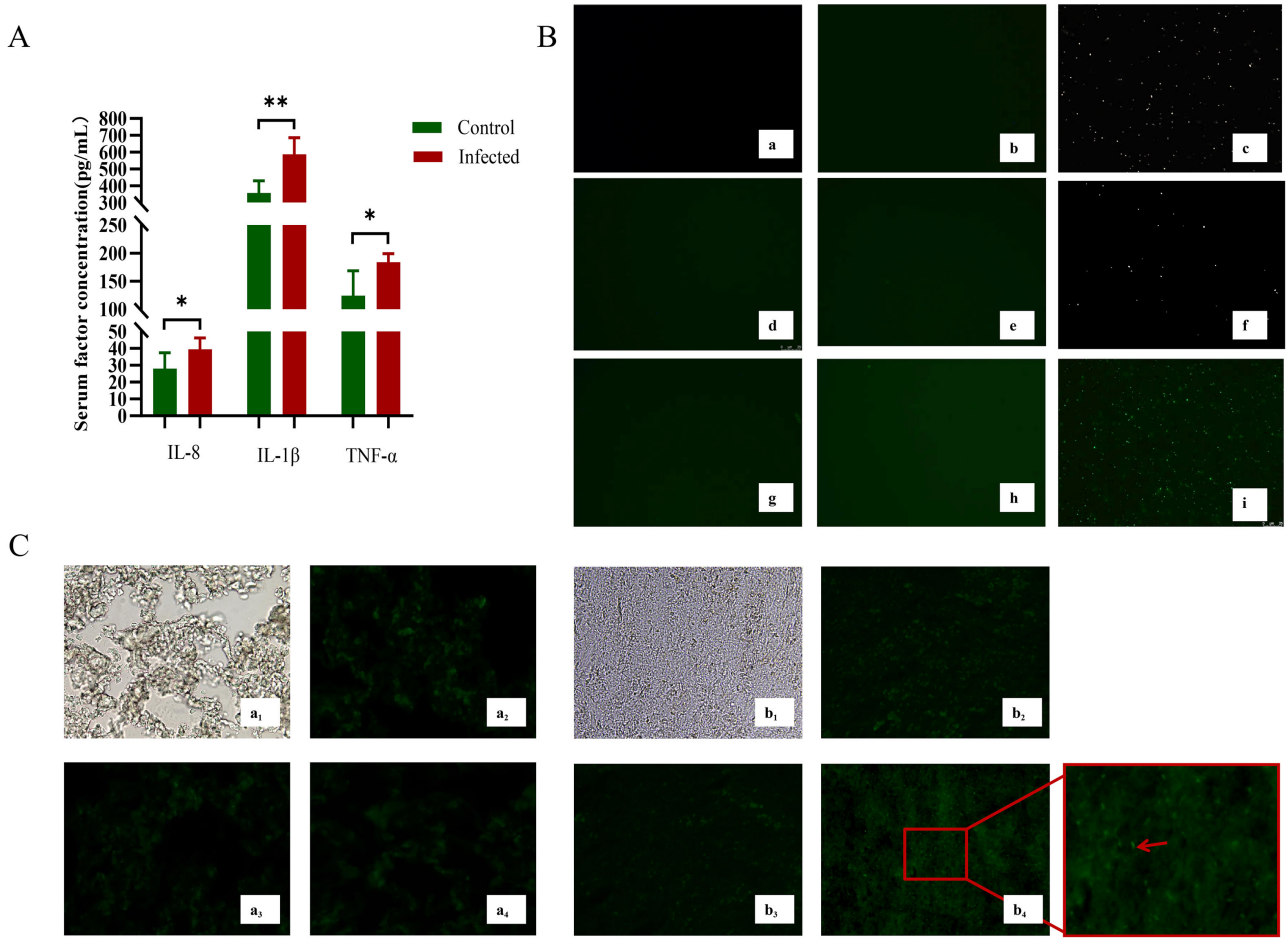
Levels of inflammatory cytokines in piglet serum and *in vivo* localization of *G. parasuis*. **(A)** Changes in the concentration of inflammatory factors in the serum.”*” P < 0.05, “**” P < 0.01. **(B)** Indirect immunofluorescence results. Note: a, d, g: indicate spontaneous fluorescence control for pleural effusion, joint effusion and pericardial effusion specimens, respectively; b, e, h: indicate pleural effusion, joint effusion and pericardial effusion fluorescent antibody control groups, respectively; c, f, i: indicate pleural effusion, joint effusion and pericardial effusion infected groups for pleural effusion fluorescence staining, respectively; Microscope magnification of 400×. **(C)** Immunofluorescence results of lung tissue. a, in the control group; b, is in the infected group; 1, Account field; 2, Specimen autofluorescence control; 3, Fluorescent antibody control; 4, Control lung; Microscope magnification of 400×.

Consistent with the observed pathology, serum levels of pro-inflammatory cytokines (TNF-α, IL-1β, and IL-8) were significantly elevated in the infected group compared to the uninfected control group (**Figure 2A**). Immunofluorescence assay (IFA) directly detected the presence of *G. parasuis* in pleural effusions, pericardial effusions, and lung tissues of infected piglets (**Figures 2B, C**). In contrast, *G. parasuis* was not detected by IFA in joint effusions from the same animals.

### 3.2 *G. parasuis* infection alters the composition and function of the lung microbiota

**Figure 3:**
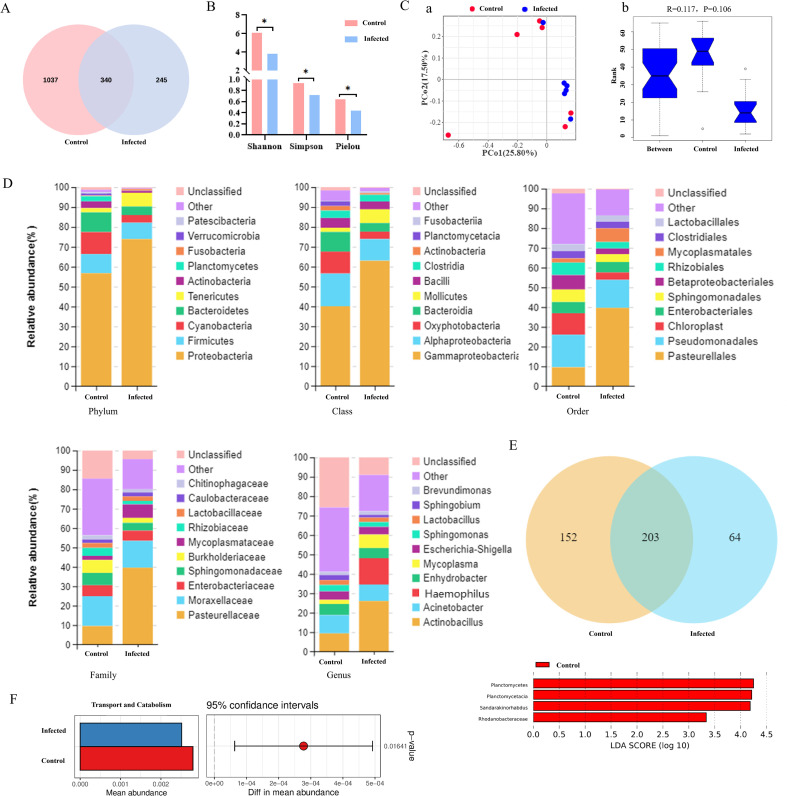
Lung microbiome profiling. **(A)** Number of OTU Venn diagram. **(B)** Alpha diversity analysis. “*” P < 0.05 indicates a significant difference between the infected group and the control group. **(C)** Beta Diversity analysis. a, PCoA scatter plot; b, ANOSIM box line plot. **(D)** Column stacking of relative abundance of lung flora. **(E)** Differential species analysis. a, Venn diagram of species at the genus level; b, LEfSe marker species analysis. **(F)** KEGG T-test difference histogram.

The composition of the lung microbiota was characterized using 16S rRNA gene sequencing. A total of 1,622 operational taxonomic units (OTUs) were identified, with 340 OTUs shared between the infected and uninfected groups (**Figure 3A**). Alpha diversity indices (Shannon, Simpson, and Pielou) were significantly lower in infected piglets, confirming a reduction in lung microbial diversity post-infection (**Figure 3B**). Beta diversity analysis (ANOSIM, R = 0.117) suggested a separation between groups, although this did not reach statistical significance (P = 0.106) (**Figure 3C**).

At the phylum level, infection led to an increase in the relative abundance of Proteobacteria and slight decreases in Firmicutes, Cyanobacteria, and Bacteroidota (**Table 1**). At the genus level, significant increases in the relative abundances of *Actinobacillus* and *Glaesserella* were observed in infected lungs (**Figure 3D**). Concurrently, the number of detectable genera was reduced post-infection. The genus *Sandarakinorhabdus* was markedly enriched in the lungs of uninfected piglets (**Figure 3E**).

**Table 1:** Table of relative abundance of the pulmonary portal level flora.

Group	Proteobacteria	Firmicutes	Cyanobacteria	Bacteroidetes
Control	56.77%	9.62%	11.05%	9.91%
Infected	73.93%	8.21%	3.89%	4.27%

“*” P < 0.05 indicates significant difference between the infected group and the control group.

Functional prediction based on 16S data revealed that the abundance of genes associated with microbial transport and catabolism was significantly lower in the lung microbiota of infected piglets (**Figure 3F**).

### 3.3 *G. parasuis* infection alters gut microbial diversity, composition, and function

**Figure 4:**
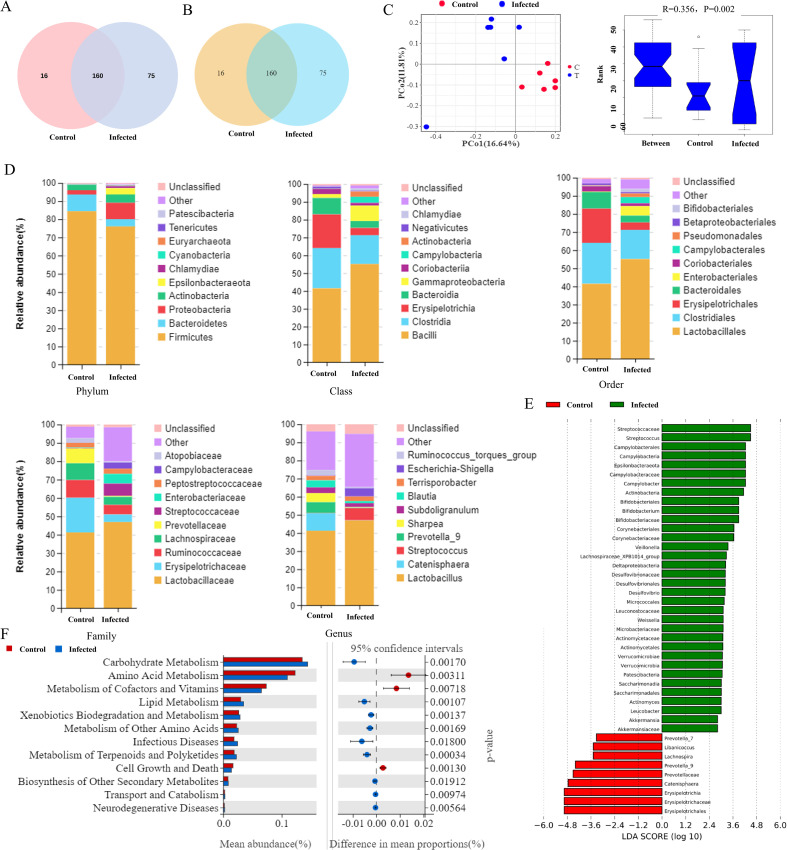
Intestinal microbiome profiling. **(A)** OTU Venn diagram. **(B)** Venn diagram of species at the genus level. **(C)** Beta Diversity analysis results. a, PCoA scatter plot; b, ANOSIM box line plot. **(D)** Column stacking diagram of relative abundance of intestinal flora. **(E)** Enriched gut microbiota at the genus level (LDA>2, P < 0.05). **(F)** KEGG T test difference histogram.

Analysis of 16S rRNA gene sequencing data revealed a total of 1,025 OTUs in cecal contents, with 398 OTUs shared between infected and uninfected piglets (**Figure 4A**). Alpha diversity indices (Chao and ACE) showed an increasing trend in infected piglets, indicating elevated microbial richness, though these changes were not statistically significant. Goods coverage values confirmed sufficient sequencing depth. Beta diversity analysis (PCoA) revealed distinct clustering between groups, which was supported by a significant ANOSIM result (R = 0.356, P<0.01) (**Figures 4B, C**).

At the phylum level, the gut microbiota was dominated by Firmicutes, Bacteroidota, Proteobacteria, and Actinobacteria. Infection led to a significant decrease in the relative abundance of Firmicutes (uninfected: 84.41%; infected: 76.10%) and Bacteroidota (9.22% to 3.93%), accompanied by an increase in Proteobacteria (2.40% to 9.08%) and Actinobacteria (2.98% to 4.54%) (**Figure 4D**; **Table 2**).

**Table 2:** Table of relative abundance of intestinal facultative level flora.

Group	Firmicutes	Bacteroidetes	Proteobacteria	Actinobacteria
Control	84.41%	9.22%	2.40%	2.98%
Infected	76.10%	3.93%	9.08%	4.54%

LEfSe analysis (LDA > 2, P<0.05) identified a pronounced dysbiosis at the genus level, characterized by a marked increase in potentially pathogenic genera and a concurrent decrease in several beneficial taxa. Specifically, infected piglets showed a significant increase (P<0.01) in the relative abundances of *Streptococcus*, *Campylobacter*, and *Desulfovibrio* genera, which are often associated with intestinal inflammation or dysfunction. In contrast, the abundances of beneficial genera such as *Prevotella_9* and *Lachnospira*, which are involved in fiber fermentation and short-chain fatty acid production, were significantly reduced (P<0.05). Interestingly, the abundance of another beneficial genus, *Bifidobacterium*, was significantly higher in the infected group (P<0.01) (**Figure 4E**; **Table 3**).

**Table 3:** Species significantly enriched at the genus level in intestinal flora.

Genus	Group	LDA score	P value
Catenisphaera	Control	4.7620	0.0039
Prevotella_9	Control	4.3950	0.0039
Lachnospira	Control	3.4856	0.0039
Libanicoccus	Control	3.4838	0.0039
Prevotella_7	Control	3.3247	0.0039
Streptococcus	Infected	4.4910	0.0039
Campylobacter	Infected	4.2357	0.0039
Bifidobacterium	Infected	3.8901	0.0039
Veillonella	Infected	3.3485	0.0039
Lachnospiraceae_XPB1014_group	Infected	3.2651	0.0033
Desulfovibrio	Infected	3.2036	0.0039
Weissella	Infected	3.1024	0.0039
Actinomyces	Infected	2.9996	0.0037
Leucobacter	Infected	2.9993	0.0039
Akkermansia	Infected	2.8167	0.0021

Functional prediction (Tax4Fun2) indicated significant alterations in 12 KEGG pathways. Pathways for Amino Acid Metabolism, Metabolism of Cofactors and Vitamins, and Cell Growth and Death were significantly downregulated in infected piglets (P<0.01). In contrast, pathways for Carbohydrate Metabolism, Lipid Metabolism, and Xenobiotics Biodegradation and Metabolism were significantly upregulated (P<0.05 or P<0.05) (**Figure 4F**; **Table 4**).

**Table 4:** The differences in relative abundance of functional genes in gut microbiota in each pathway.

Pathway	Control	Infected	P-value	Change	Significant
Carbohydrate Metabolism	0.1348	0.1443	0.0017	Up	**
Amino Acid Metabolism	0.1229	0.1093	0.0031	Down	**
Metabolism of Cofactors and Vitamins	0.0734	0.0649	0.0072	Down	**
Lipid Metabolism	0.0294	0.0345	0.0011	Up	**
Xenobiotics Biodegradation and Metabolism	0.0261	0.0283	0.0014	Up	**
Metabolism of Other Amino Acids	0.0226	0.0253	0.0017	Up	**
Infectious Diseases	0.0180	0.0242	0.0180	Up	*
Metabolism of Terpenoids and Polyketides	0.0182	0.0222	0.0003	Up	**
Cell Growth and Death	0.0163	0.0134	0.0013	Down	**
Biosynthesis of Other Secondary Metabolites	0.0076	0.0082	0.0191	Up	*
Transport and Catabolism	0.0019	0.0022	0.0097	Up	**
Neurodegenerative Diseases	0.0015	0.0018	0.0056	Up	**

“*” P < 0.05 represents significant differences between two groups. “**” P < 0.01 represents extremely significant differences between two groups.

### 3.4 *G. parasuis* infection perturbs the arginine biosynthesis pathway and alters key metabolites in cecal contents

**Figure 5:**
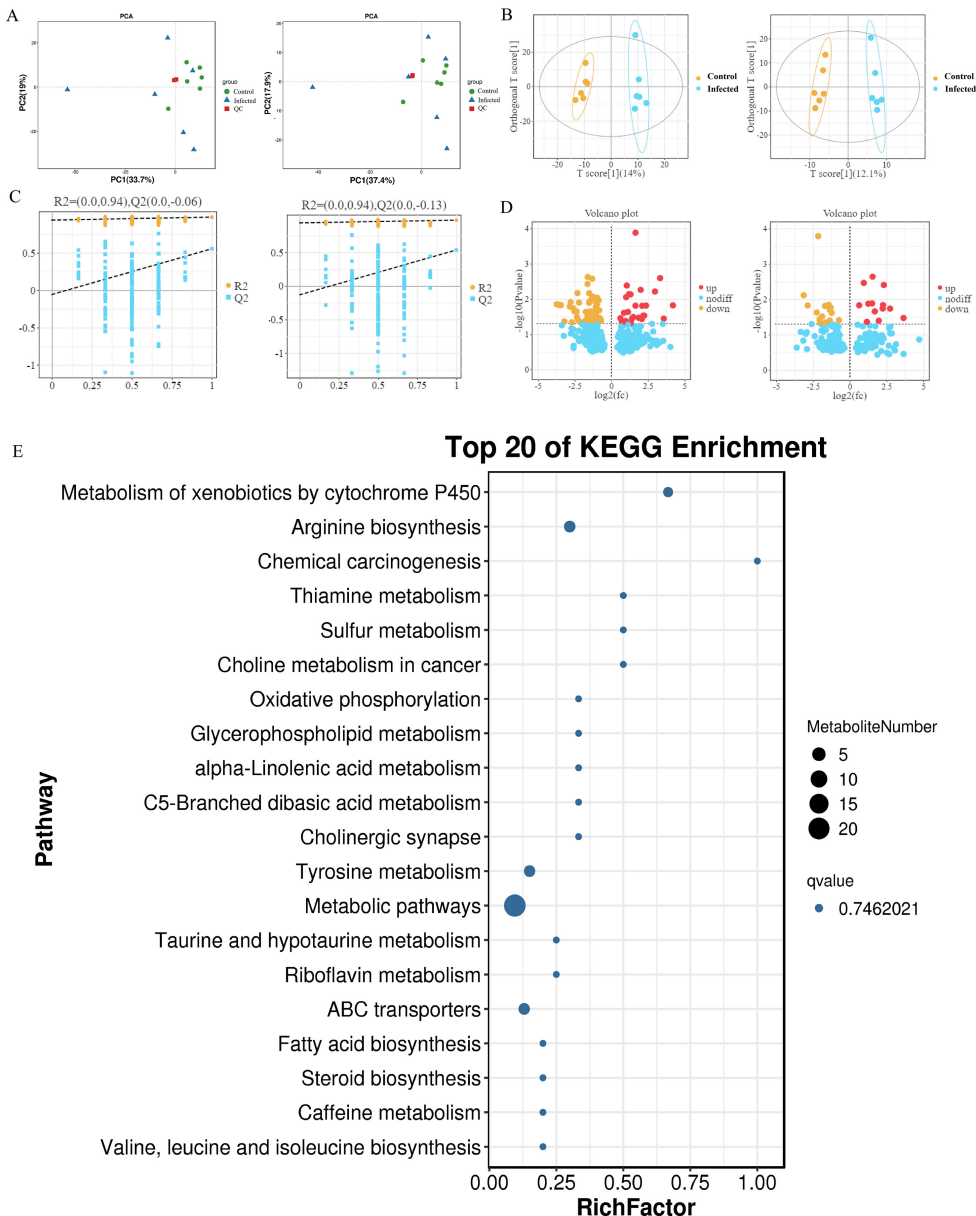
Intestinal content metabolite profiling. **(A)** Principal component analysis of QC samples. **(B)** OPLS-DA score graph. **(C)** OPLS-DA substitution test. **(D)** Volcano map of differential metabolites. In **(A–D)**, the left diagram is in positive ion mode, and the right diagram is in negative ion mode. **(E)** The top 20 metabolic pathways KEGG enrichment. The vertical coordinate is the pathway and the horizontal coordinate is the enrichment factor, which is the proportion of differential metabolites in that pathway to the total metabolites in that pathway.

Untargeted metabolomic analysis of cecal contents using both positive and negative ionization modes showed clear separation between infected and uninfected piglets in the OPLS-DA score plots, with robust model validation (**Figures 5A–C**).

A total of 119 differential metabolites were identified (VIP ≥ 1, P<0.05). Among these, 30 metabolites (22 in positive and 8 in negative mode) were successfully annotated in the KEGG database (**Figure 5D**). Fifteen metabolites, including liquiritigenin (P<0.01), citraconic acid (P<0.05), and argininosuccinic acid (P<0.05), were significantly decreased in infected piglets. Conversely, fifteen metabolites, including citrulline (P<0.01) and N-acetyl-L-phenylalanine (P<0.05), were significantly increased (**Table 5**).

**Table 5:** Information related to differential metabolites.

Name	Formula	P value	Vip	Mode	Group	C_ID
Liquiritigenin	C15H12O4	0.0002	2.5310	NEG	Control	C09762
Citraconic acid	C5H6O4	0.0172	2.0149	NEG	Control	C02226
Cyclamic acid	C6H13NO3S	0.0197	1.9342	NEG	Control	C02824
Capric acid	C10H20O2	0.0235	1.8459	NEG	Control	C01571
Taurine	C2H7NO3S	0.0438	1.6398	NEG	Control	C00245
Ergocalciferol	C28H44O	0.0037	1.9869	POS	Control	C05441
Argininosuccinic acid	C10H18N4O6	0.0105	1.9459	POS	Control	C03406
N-Acetylornithine	C7H14N2O3	0.0127	1.9390	POS	Control	C00437
Riboflavin-5-phosphate	C17H21N4O9P	0.0123	1.9179	POS	Control	C00061
L-Adrenaline	C9H13NO3	0.0123	1.9175	POS	Control	C00788
Deoxyguanosine	C10H13N5O4	0.0209	1.8009	POS	Control	C00330
2-Oxindole	C8H7NO	0.0360	1.7719	POS	Control	C12312
Pregnenolone	C21H32O2	0.0351	1.6212	POS	Control	C01953
DL-Panthenol	C9H19NO4	0.0483	1.5356	POS	Control	C05944
Xanthine	C5H4N4O2	0.0424	1.5057	POS	Control	C00385
Citrulline	C6H13N3O3	0.0039	2.1641	NEG	Infected	C00327
N-Acetyl-L-phenylalanine	C11H13NO3	0.0130	2.1529	NEG	Infected	C03519
Cytidine-5’-monophosphate	C9H14N3O8P	0.0434	1.7410	NEG	Infected	C00055
2-(1H-indol-3-yl) acetic acid	C10H9NO2	0.0041	2.0491	POS	Infected	C00954
4-Methyl-5-thiazoleethanol	C6H9NOS	0.0152	1.9502	POS	Infected	C04294
Indole-5,6-quinone	C8H5NO2	0.0155	1.9205	POS	Infected	C05579
3-Succinoylpyridine	C9H9NO3	0.0401	1.7644	POS	Infected	C19569
4-Guanidinobutanoic acid	C5H11N3O2	0.0443	1.7067	POS	Infected	C01035
Dopaquinone	C9H9NO4	0.0244	1.6917	POS	Infected	C00822
D-Raffinose	C18H32O16	0.0316	1.6906	POS	Infected	C00492
Carnosine	C9H14N4O3	0.0339	1.6742	POS	Infected	C00386
Jasmonic acid	C12H18O3	0.0326	1.5927	POS	Infected	C08491
2-Aminobenzen-Esulfonic acid	C6H7NO3S	0.0407	1.5328	POS	Infected	C06333
2-Naphthol	C10H8O	0.0458	1.4601	POS	Infected	C11713
Choline	C5H13NO	0.0467	1.4249	POS	Infected	C00114

POS is in positive ion mode, NEG is in negative ion mode.

KEGG pathway enrichment analysis of the 30 annotated differential metabolites revealed significant alterations in specific metabolic pathways. The top 20 most enriched pathways are listed in **Table 6**, with Metabolism of xenobiotics by cytochrome P450 and Arginine biosynthesis showing the most significant enrichment (both P<0.05, **Figure 5E**). Notably, three key intermediates of the arginine biosynthesis pathway (argininosuccinate, N-acetylornithine, and citrulline) were identified among the differential metabolites.

**Table 6:** Differential metabolic pathways.

KEGG pathway	P value	Q-value	Pathway ID
Metabolism of xenobiotics by cytochrome P450	0.020448	0.746202	ko00980
Arginine biosynthesis	0.045935	0.746202	ko00220
Chemical carcinogenesis	0.086207	0.746202	ko05204
Thiamine metabolism	0.165209	0.746202	ko00730
Sulfur metabolism	0.165209	0.746202	ko00920
Choline metabolism in cancer	0.165209	0.746202	ko05231
Oxidative phosphorylation	0.237590	0.746202	ko00190
Glycerophospholipid metabolism	0.237590	0.746202	ko00564
alpha-Linolenic acid metabolism	0.237590	0.746202	ko00592
C5-Branched dibasic acid metabolism	0.237590	0.746202	ko00660
Cholinergic synapse	0.237590	0.746202	ko04725
Tyrosine metabolism	0.242523	0.746202	ko00350
Metabolic pathways	0.275133	0.746202	ko01100
Taurine and hypotaurine metabolism	0.303886	0.746202	ko00430
Riboflavin metabolism	0.303886	0.746202	ko00740
ABC transporters	0.317148	0.746202	ko02010
Fatty acid biosynthesis	0.364594	0.746202	ko00061
Steroid biosynthesis	0.364594	0.746202	ko00100
Caffeine metabolism	0.364594	0.746202	ko00232
Valine, leucine and isoleucine biosynthesis	0.364594	0.746202	ko00290

### 3.5 Correlation analysis between altered gut microbiota and metabolites

**Figure 6:**
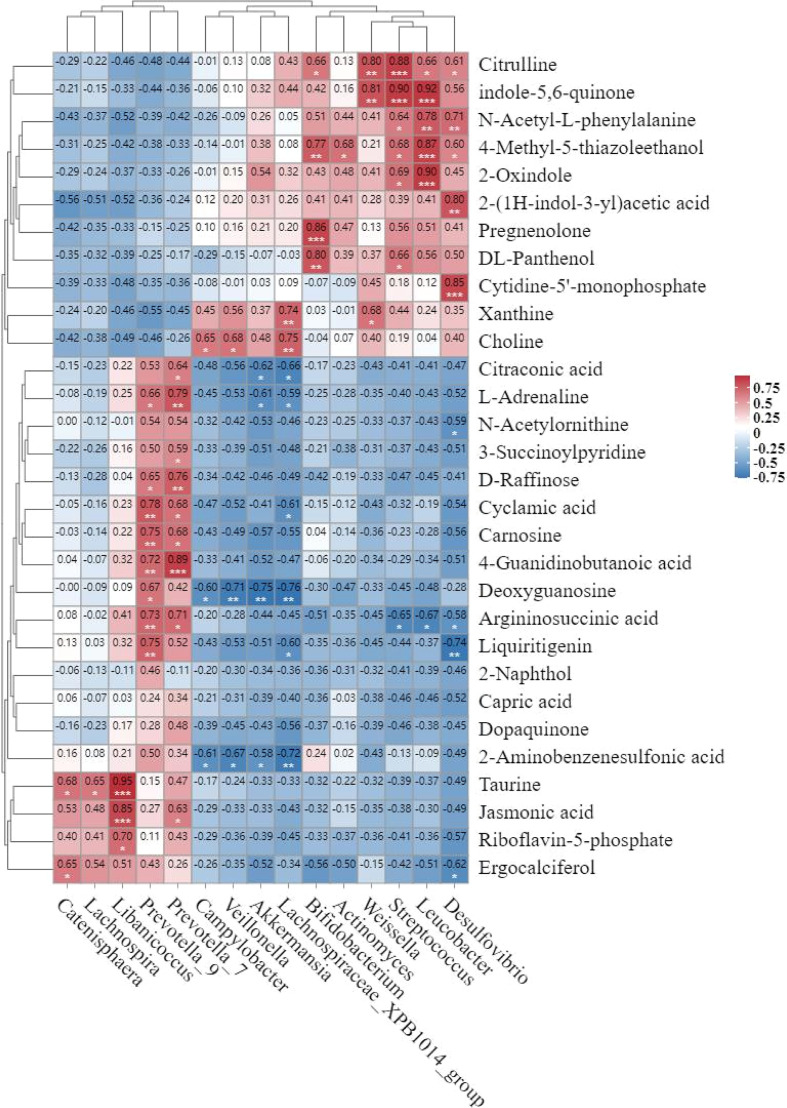
Heat map of correlation between differential flora and differential metabolites. Red represents correlation r>0, which is positive; blue represents correlation r<0, which is negative; “*” P < 0.05, “**” P < 0.01, “***” P < 0.001.

Pearson correlation analysis between the 15 significantly altered gut microbial genera and the 30 differential metabolites identified a network of 71 statistically significant associations (P<0.05, **Figure 6**). Notably, *Desulfovibrio*, *Prevotella_7*, *Prevotella_9*, and *Lachnospiraceae_XPB1014_group* each exhibited significant correlations with more than eight differential metabolites, while *Streptococcus* and *Leucobacter* correlated with seven and six metabolites, respectively.

Of particular relevance to the perturbed arginine biosynthesis pathway, two of its key intermediates showed specific microbial correlations. Argininosuccinate levels were negatively correlated with *Leucobacter* (P<0.05), *Streptococcus* (P<0.05), and *Desulfovibrio* (P<0.05), but positively correlated with *Prevotella_9* (P<0.01). N-Acetylornithine exhibited a negative correlation specifically with *Desulfovibrio* (P<0.05).

### 3.6 *G. parasuis* infection induces intestinal structural damage and impairs barrier function

**Figure 7:**
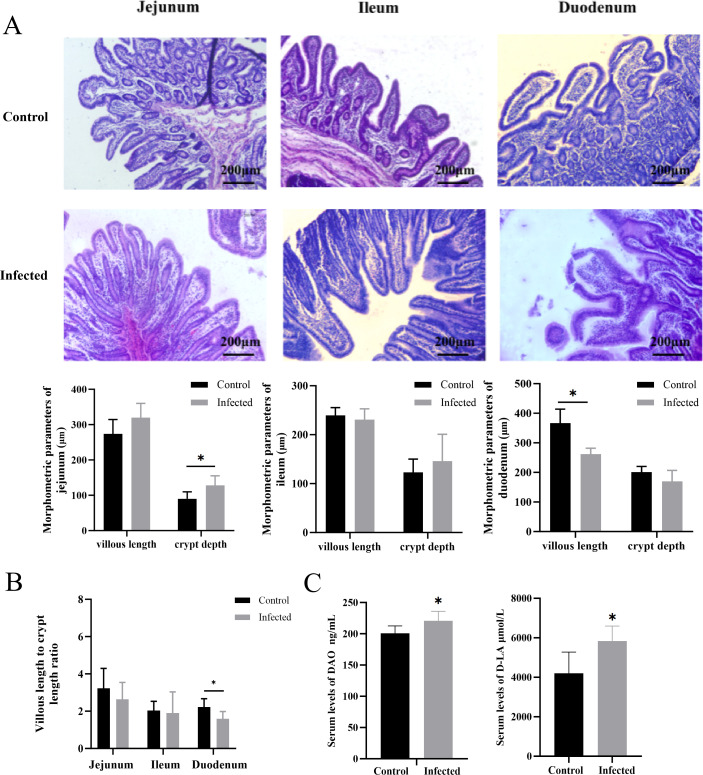
Intestinal barrier function measurement results. **(A)** Pathology tissue testing. Morphological results of the small intestine and statistical graph of small intestine data. **(B)** The villus-to-crypt (V/C) ratio in the small intestine. **(C)** Serum levels of DAO and D-LA. “*” P < 0.05 indicates a significant difference between the infected group and the control group.

Histopathological analysis of the small intestine revealed disorganized intestinal epithelial cells, mild villus damage, and glandular atrophy in infected piglets compared to the well-structured mucosa of uninfected controls (**Figure 7A**). Morphometric measurements of the intestinal architecture confirmed these observations. In infected piglets, crypt depth in the jejunum was significantly increased (P<0.05), while villus length and the villus-to-crypt (V/C) ratio in the duodenum were significantly reduced (P<0.05). Similar decreasing trends in villus length and villus-to-crypt (V/C) ratio were observed in the ileum, although these did not reach statistical significance (**Figure 7B**).

Consistent with the observed structural damage, serum biomarkers of intestinal barrier integrity were significantly altered. Serum levels of diamine oxidase (DAO) and D-lactate were both significantly elevated in infected piglets (P<0.05) (**Figure 7C**), indicating increased intestinal permeability.

## 4 Discussion

Glässer’s disease is widespread globally and causes significant economic losses in the swine industry due to its high mortality rate (Lin et al., 2025; Yan et al., 2023). *G. parasuis* is the pathogen responsible for Glässer’s disease, which induces systemic fibrinous inflammation and bacterial pneumonia following infection (Guo et al., 2025; Zhou et al., 2022). While traditional research has focused on pathogen serotyping, virulence factors, and host immunity, the dynamic alterations in the host’s microbiota during infection remain poorly understood. This study established a piglet infection model with the *G. parasuis* XX0306 strain, providing the first systematic analysis of the concurrent impact of this infection on the pulmonary and intestinal microbial ecosystems, host metabolism, and intestinal barrier integrity. These findings illuminate the systemic pathology of the disease through an integrated microbiota-metabolism-immunity perspective.

The infection induced a marked restructuring of microbial communities. In the lungs, diminished diversity was accompanied by an increased relative abundance of Proteobacteria and a decrease in Firmicutes, a dysbiotic pattern analogous to that observed in chronic obstructive pulmonary disease (Dicker et al., 2021; Huang et al., 2014). This shift suggests the infection may foster a pulmonary niche favoring opportunistic pathogens (de Steenhuijsen Piters et al., 2015; Perez-Cobas et al., 2023). Corresponding functional predictions indicated a significant downregulation of genes involved in material transport and catabolic metabolism within the lung microbiota. This functional impairment likely compromises local microbial homeostasis and clearance capacity, correlating with the observed severe pulmonary inflammatory pathology.

More profound alterations were evident within the gut microbiota. A significant depletion of beneficial bacterial taxa was noted, including members of the genus *Prevotella* and the family Lachnospiraceae, which are recognized as principal producers of short-chain fatty acids such as butyrate and propionate (Fitzgerald et al., 2025; Sebastia et al., 2024). These metabolites serve as the primary energy source for colonocytes, reinforce epithelial barrier function, and facilitate the differentiation of anti-inflammatory regulatory T cells (Ploger et al., 2012; Saleri et al., 2022; Takahashi et al., 2020). Direct evidence confirms that intestinal *Prevotella* colonization helps maintain steady-state levels of SCFAs and promotes the production of the protective cytokine IL-18 in colonic epithelium, which is crucial for barrier integrity and immune tolerance (Iljazovic et al., 2021). Consequently, the depletion of these bacteria likely represents an active driver of subsequent pathophysiology, leading to SCFA deficiency, diminished intestinal immune signaling, and exacerbated barrier damage. Concurrently, an enrichment of bacteria with potential pathogenic traits was observed. The expansion of *Desulfovibrio*, a producer of epithelial-damaging hydrogen sulfide, and pro-inflammatory *Streptococcus* species may not merely be a consequence of dysbiosis but could actively drive intestinal inflammation and metabolic perturbation. KEGG pathway analysis further revealed a functional recalibration. Reduced gene abundances in amino acid and cofactor metabolism pathways may limit the supply of critical immunonutrients like arginine, while increased abundances in carbohydrate and lipid metabolism genes may reflect nutrient competition by expanding pathobionts or indicate a state of host metabolic stress. This gut dysbiosis signature, characterized by the loss of commensal symbionts and the rise of potential pathogens, mirrors patterns reported following other respiratory infections, suggesting that gut-lung axis disruption may be a common feature of respiratory pathophysiology (Hakansson et al., 2018).

Non-targeted metabolomic profiling revealed significant disturbances in the intestinal metabolome of infected piglets, with pronounced suppression of the arginine biosynthesis pathway. Levels of the metabolic precursor argininosuccinate were significantly diminished and exhibited correlations with key bacterial taxa, indicating that gut microbiota structural disruption directly modulates host arginine metabolism. Arginine metabolism constitutes a core hub linking microbial dysbiosis to immune compromise. Conventionally, arginine deficiency impairs the host by limiting substrates for nitric oxide synthesis in macrophages and for polyamine production essential for tissue repair (Ji et al., 2019; Karadima et al., 2025; Marti and Reith, 2021). Emerging evidence, however, reveals a competitive dimension to this metabolic disturbance. Pathogens such as *Streptococcus suis* can actively consume host arginine via the arginine deiminase system to fuel their own energy needs and acid resistance, while simultaneously restricting macrophage production of bactericidal nitric oxide (Zhang et al., 2024). Thus, the observed arginine metabolic inhibition likely represents a vicious cycle of host synthetic deficiency compounded by pathogen consumption. These findings provide a direct rationale for exploring arginine supplementation as a nutritional immunomodulatory strategy. Alterations in the cytochrome P450 pathway indicate a broader dysregulation of metabolic homeostasis under infectious stress, potentially amplifying systemic inflammatory responses.

Histopathological assessment and elevated serum markers of intestinal permeability confirmed significant damage to the intestinal mucosal barrier. This finding crucially links a primary respiratory infection to distal gastrointestinal dysfunction. The breach of intestinal barrier integrity can facilitate the translocation of microbial products, thereby driving a systemic inflammatory response and establishing a vicious cycle of gut leakiness and systemic inflammation (Kinashi and Hase, 2021; Panpetch et al., 2020; Zhang et al., 2024). We propose that pulmonary *G. parasuis* infection disrupts gut homeostasis via the gut-lung axis, involving a cascade of beneficial bacteria depletion, pathobiont expansion, physical barrier disruption, and subsequent microbial translocation, ultimately amplifying multi-organ injury. This pathological cycle finds strong parallel support in other disease models (Budden et al., 2017; Wypych et al., 2019). Research in chronic kidney disease has conclusively demonstrated that uremia-induced gut dysbiosis and barrier dysfunction lead to translocation of viable bacteria and endotoxin into the circulation, directly driving persistent systemic inflammation (Vaziri et al., 2016). Critically, eradication of the dysbiotic microbiota with antibiotics fully reverses all inflammatory markers, establishing a causal relationship (Andersen et al., 2017). This study provides initial evidence for the existence of a similar integrated pathway in *G. parasuis* pathogenesis.

The present work primarily establishes correlations between microbial shifts and metabolic alterations. To advance beyond association and establish causality, future investigations should employ metagenomic sequencing for higher-resolution functional profiling and conduct targeted quantification of key metabolites, including short-chain fatty acids and arginine pathway intermediates. *In vitro* models utilizing intestinal organoids or epithelial-immune cell co-cultures would be valuable for directly testing the effects of specific bacterial taxa or their metabolic products on barrier function and immune activation. Finally, interventional animal studies evaluating the therapeutic potential of butyrate, arginine, or defined probiotic consortia against *G. parasuis* infection are essential to translate these mechanistic insights into practical strategies for disease control.

## Data Availability

The datasets presented in this study can be found in online repositories. The names of the repository/repositories and accession number(s) can be found below: https://www.ncbi.nlm.nih.gov/, PRJNA1283987 https://www.ebi.ac.uk/metabolights/, MTBLS12802.
